# TREM2 in neurodegeneration and diseases

**DOI:** 10.1038/s41380-026-03505-7

**Published:** 2026-03-06

**Authors:** Altaf A. Abdulkhaliq, Glowi Alasiri, Bonglee Kim, Johra Khan, Amir Ajoolabady, Shimaa Mohammad Yousof, Jun Ren, Jaakko Tuomilehto, Anwar Borai, Bahauddeen M. Alrfaei, Domenico Pratico

**Affiliations:** 1 Department of Biochemistry, Faculty of Medicine, Umm Al-Qura University, Mecca, Saudi Arabia; 2 Department of Biochemistry, College of Medicine, Imam Mohammad Ibn Saud Islamic University (IMSIU), Riyadh, 13317 Saudi Arabia; 3 Department of Pathology, College of Korean Medicine, Kyung Hee University, Hoegidong Dongdaemun-gu, Seoul, 02447 Republic of Korea; 4 Department of Medical Laboratory Sciences, College of Applied Medical Sciences, Majmaah University, Al Majmaah, 11952 Saudi Arabia; 5 National Clinical Research Center for Interventional Medicine, Shanghai, 200032 China; 6 State Key Laboratory of Cardiovascular Diseases, Zhongshan Hospital, Fudan University, Shanghai, 200032 China; 7 Department of Physiology, Faculty of Medicine, King Abdulaziz University, Jeddah, Saudi Arabia; 8 Department of Physiology, Faculty of Medicine, Suez Canal University, Ismailia, Egypt; 9 Neuroscience and Geroscience Unit, King Fahad Medical Research Centre, King Abdulaziz University, Jeddah, Saudi Arabia; 10 Health Promotion Unit, Finnish Institute for Health and Welfare, Helsinki, Finland; 11 King Abdullah International Medical Research Center (KAIMRC), King Saud Bin Abdulaziz University for Health Sciences (KSAU-HS), King Abdulaziz Medical City, Ministry of National Guard Health Affairs, Jeddah, Saudi Arabia; 12 King Abdullah International Medical Research Center (KAIMRC)/ King Saud Bin Abdulaziz University for Health Sciences (KSAU-HS), Riyadh, 11426 Saudi Arabia; 13 Department of Neural Sciences, Lewis Katz School of Medicine, Temple University, Philadelphia, PA 19140 USA

**Keywords:** Neuroscience, Cell biology, Psychiatric disorders

## Abstract

Triggering receptor expressed on myeloid cells 2 (TREM2) is a cell surface transmembrane receptor from the TREM receptor family, predominantly expressed on the microglia in the central nervous system (CNS). TREM2-initiated signaling plays a crucial role in regulating neuroinflammation and neurodegeneration, particularly in the context of neurodegenerative diseases such as Alzheimer’s disease (AD) and Parkinson’s disease (PD), through the activation of downstream signaling pathways and transcriptional regulation of relevant genes. In this review, we aim to provide a concise review of the role and mechanistic implications of TREM2 in neurodegeneration and neuroinflammation, with a specific focus on AD and PD. We will discuss the most recent preclinical studies to highlight current advancements in the field. This review is intended to support both basic researchers and clinicians by enhancing their understanding of microglial function in the pathophysiology of AD and PD, as well as its role in neuroinflammation and neurodegeneration. Ultimately, we hope this contribution will pave the way for new discoveries and the development of potential therapeutic interventions.

## Introduction: an overview of neurodegernation and diseases

Neurodegeneration is characterized by progressive loss of neurons and neuronal cell death, including both apoptotic and necrotic pathways, in key brain regions associated with motor and cognitive dysfunctions [1–5]. The underlying causes of neurodegeneration are multifactorial, encompassing genetic factors, endogenous processes, and environmental stimuli. These factors often interact with the aging process through complex and poorly understood mechanisms [6–8]. Additionally, various medical conditions such as stroke, traumatic brain injuries, and brain tumors (e.g., malignant gliomas), as well as alcoholism, contribute to neurodegeneration. Neurotoxicity induced by exposure to toxic chemicals, environmental pollutants or viral infections also plays a significant role [9–13]. Globally, neurodegenerative diseases [14, 15] are on the rise, manifesting in various forms, with AD and PD being the most prevalent and clinically significant subsets [16–19].

AD is a progressive neurodegeneration characterized by formation of interneuronal amyloid-β (Aβ) plaques and intraneuronal neurofibrillary tau tangles, leading to synaptic dysfunction, neuronal cell death, ultimately, cognitive impairment and dementia [20–22]. Physiologically, Aβ peptides elicit multiple functions such as governance of synaptic function, neuronal growth and survival, as well as protection against oxidative damage, neurotoxic compounds, and pathogens [23–26]. These peptides are ultimately destined for removal and clearance via multiple mechanisms such as enzyme-based degradation [27]. Failure in these mechanisms as well as increased or abnormal production of amyloid precursor protein (APP) all could amplify Aβ level, causing its accumulation and oligomerization in neurons and subsequent spread to the interneuronal space, ultimately, forming Aβ plaques that in the vicinity of synapses may cause synaptic dysfunction via diverse mechanisms such as altering glutamate receptors, forming pore-like channels, modulating signaling cascades, and inducing mitochondrial dysfunction in neurons [27–29].

As the second most prevalent subset of neurodegenerative diseases, PD affects almost 1–2% of elderlies aged 60 years or over [30–33]. Phenotypically, PD is accompanied by both motor symptoms and movement disorders such as muscular rigidity, bradykinesia, and resting tremors, as well as non-motor such as neuropsychiatric symptoms (e.g., anxiety, depression) and gastrointestinal symptoms (e.g., constipation, dysphagia) [19, 34–37]. PD pathogenesis involves aberrant accumulation of misfolded soluble alpha-synuclein (α-Syn) monomers in brain parenchyma [38], which subsequently transform into oligomers to be aggregated into mature insoluble fibrils, and ultimately forming intraneuronal inclusions such as Lewy bodies (LBs) and Lewy neurites (LNs) [39–41]. Physiologically, α-Syn serves as a negative regulator of dopamine release in presynaptic neurons; therefore, contributing to neural plasticity and synaptic function [42, 43]. α-Syn is mainly expressed in the substantia nigra, the hippocampus, and the cortex with α-helical conformation before oligomerization into neurotoxic plaques [39, 44]. Although the main cause of α-Syn misfolding in PD remains elusive, multiple factors including post-translational modifications (e.g., phosphorylation of serine residues), oxidative stress, genetic mutations, mitochondrial dysfunction, impaired protein clearance mechanisms, and environmental stimuli may contribute to abnormal folding and aggregation of α-Syn [45–49].

## Neuroinflammation and its link with neurodegeneration

In the CNS, neuroinflammation manifests as a chronic, unresolved inflammatory response that can lead to various brain anomalies and conditions [50–55]. A wide range of CNS immune cell types including resident glial cells (e.g., astrocytes, oligodendrocytes, microglia), brain-associated macrophages (BAMs), and peripheral immune cells, as well as non-immune cells such as endothelial cells participate in the initiation and regulation of neuroinflammation [56, 57]. Upon activation, these cells release diverse immune mediators, including chemokines, cytokines, proteases, and growth factors, which modulate different levels of neuroinflammation within the CNS [50, 58, 59]. Nonetheless, microglia are the CNS-resident macrophages and primary players in regulation of neuroinflammation [60–65]. Although neuroinflammation could bring various pathological consequences in CNS, one of the key events is the link between neuroinflammation and neurodegeneration [66] and development/pathophysiology of AD and PD [1, 67–69]. In return, both AD and PD are accompanied by proinflammatory responses that exacerbate neuroinflammation in a vicious cycle [67, 70, 71]. Both exogenous and endogenous stimuli such as infections, autoimmunity, metabolic stress, neoplasm, trauma, ischemia, and degeneration could drive neuroinflammation by provoking the release of proinflammatory chemokines and cytokines from the CNS immune and non-immune cells [50, 54, 59, 72, 73].

Generally, protein aggregates in neurodegenerative diseases trigger neuroinflammation, to exacerbate protein aggregation and neurodegeneration [1]. Although the intricate link between neuroinflammation and neurodegeneration could be multifaceted and complicated [1], in one example, Aβ plaques induce activation of microglia, which release cytokines, leading to activation of A1 astrocytes [74, 75]. Subsequently, A1 astrocytes secrete a substantial volume of pro-inflammatory factors escalating neuroinflammation and a neurotoxin inducing neuronal and oligodendrocyte death thus exacerbating neurodegernation [75, 76]. Some prominent reviews have comprehensively elucidated the link between neuroinflammation and neurodegeneration [1, 70, 71].

## TREM2: biology and function

**Fig. 1:**
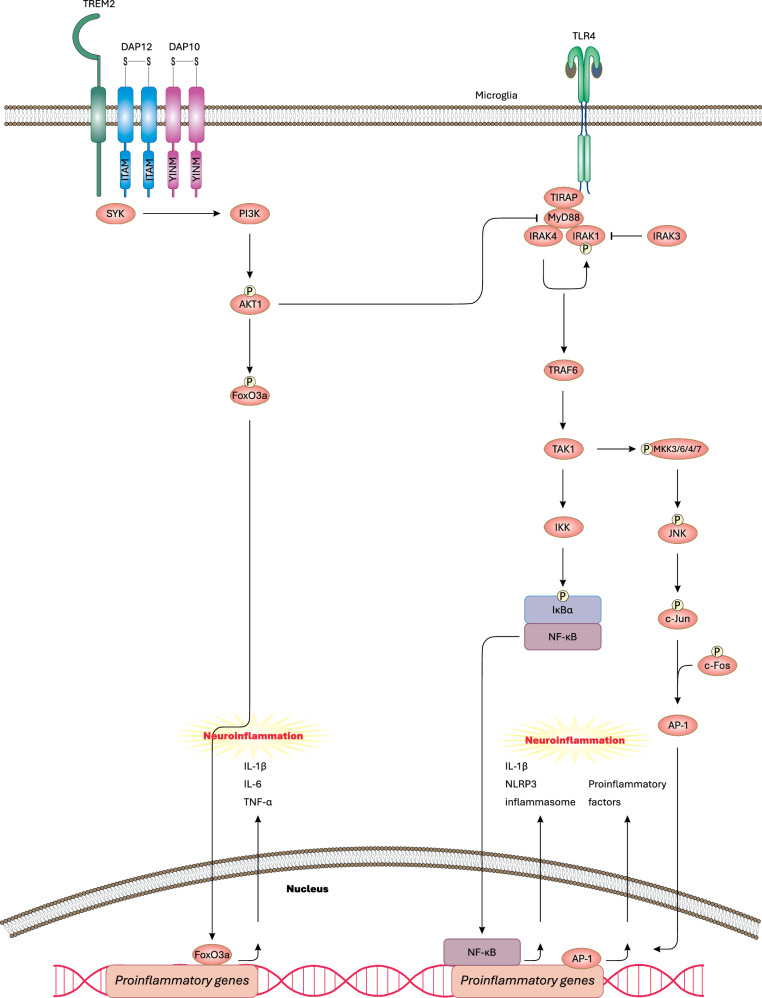
Microglia TREM2 and associated downstream signaling. DAP10 (DNAX activating protein of 10 kDa) and DAP12 (DNAX activating protein of 12 kDa) are two adaptor proteins that transduce TREM2 signaling. Mechanistically, DAP12 recruits SYK (spleen associated tyrosine kinase) through ITAM (immunoreceptor tyrosine-based activation motif) motifs, in turn, SYK binds to and activates PI3K. DAP10 also contributes to further recruitment of PI3K, which phosphorylates and activates AKT1. Activated AKT1 can regulate FoxO3a transcription factor, leading to inactivation/repression of proinflammatory genes. IRAK3 (interleukin 1 receptor associated kinase 3) and AKT1 can also interfere with TLR4 signaling by blocking IRAK4/l (interleukin 1 receptor associated kinase 4/1) and MyD88 (myeloid differentiation primary response 88), respectively, resulting in inhibition of MAPK and NF-κB signalings and resolution of neuroinflammation. IκBα inhibitor of nuclear factor kappa B, IKK IκB kinase complex, TAK1 mitogen-activated protein kinase kinase kinase 7.

TREM2 is a 230 amino acid-containing transmembrane glycoprotein receptor with a V-immunoglobulin extracellular domain and a cytosolic domain [77]. Additionally, two adaptor proteins including DAP10 (DNAX-activating protein 10) and DAP12 (DNAX activating protein of 12 kDa) mediate TREM2 intracellular signaling (Fig. 1) [78–81]. TREM2 is exclusively expressed in the microglia, particularly in the white matter, spinal cord, and the hippocampus [78, 81–83]. Functionally, the expression of TREM2 is upregulated under brain’s pathological states such as AD and PD, stroke, hemorrhage, and trauma thus serving as a neuroprotective mechanism [82, 84–91]. Thus, unsurprisingly, *TREM2* gene variants including *R47H* and *R62H* are linked with increased AD risk [92].

Abundant evidence supports anti-inflammatory role of TREM2 in microglia; thus its activation leads to suppression of neuroinflammation in the CNS [93, 94]. In compliance, anti-inflammatory cytokines including interleukin-13 (IL-13) and interleukin-4 (IL-4) induce upregulation of *TREM2* expression, whereas pro-inflammatory factors such as tumor necrosis factor alpha (TNF-α) and interleukin-1 beta (IL-1β) induce its downregulation [93, 95–100]. Alternatively, activation of TREM2 promotes phagocytic ability of microglia, which enhances the removal of Aβ plaques and apoptotic neurons, thereby contributing to neuroprotection [92, 101, 102]. Another neuroprotective mechanism of TREM2 involves its interaction with microglial heparan sulfate on the plasma membrane, forming a complex that facilitates the uptake of ApoE3 (apolipoprotein E3) – a neuroprotective module [103–108]. Notably, ApoE variants (e.g., Apoe4 vs Apoe3) may interact differentially with TREM2, potentially leading to divergent functional outcomes [109, 110]. However, these variant-specific interactions remain insufficiently explored in the current literature. Given the well-established link between neuroinflammation and neurodegeneration, elucidating the role of TREM2 in regulating neuroinflammatory responses may also provide critical insights into its broader contribution to neurodegenerative processes.

## TREM2 in Alzheimer’s associated neuroinflammation and neurodegeneration

**Fig. 2:**
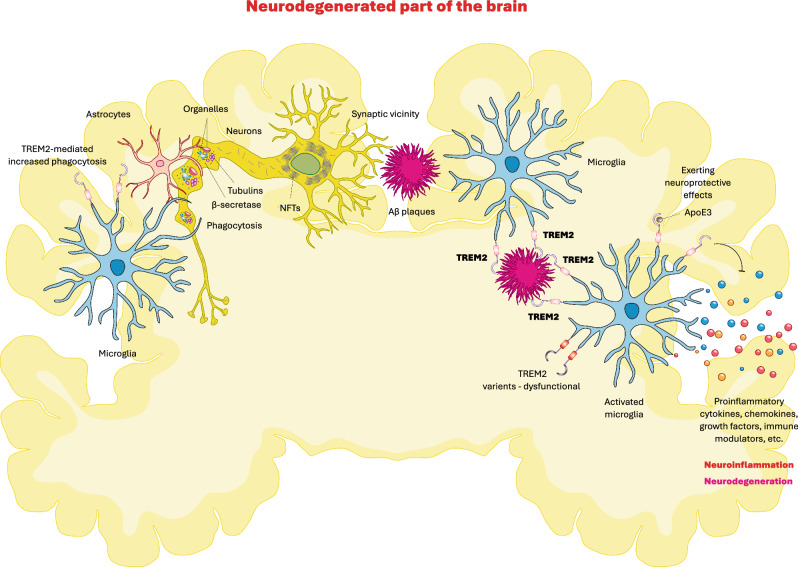
Microglia-mediated neuroprotection through TREM2. A β plaques bind to and interact with microglia TREM2, leading to activation of microglia response, increased phagocytosis, and inhibition of neuroinflammation. Moreover, TREM2 promotes ApoE3 uptake thus exerting additional neuroprotective effects. Nonetheless, *TREM2* variants are closely linked with increased AD susceptibility.

Current evidence suggests that microglia TREM2 binds to and interacts with oligomeric Aβ forms such as Aβ_42_ oligomers, thereby activating TREM2 signaling and subsequent response of microglia to AD and Aβ pathology [82, 111–113]. Alternatively, Aβ oligomers could be internalized into microglia through interaction with TREM2 and elicit downstream signalings [82, 111–113]. However, the identity and biological/pathological impact of these signalings have not been fully elucidated, hence, the interaction between microglia TREM2 and Aβ plaques could be the subject of future research for further discoveries (Fig. 2).

Recently, it was shown that activation of microglia TREM2 induced upregulation of several complement system genes, as well as antigen presentation, phagosomal, and lysosomal genes, all contributing to improved clearance of Aβ plaques in mice with early and middle stage AD [114]. Additionally, TREM2 interacted with Aβ subtypes thus forming compact plaque cores whose biological/pathological implication still remains unknown but supposedly may serve as a mechanism to promote Aβ clearance [114]. To this end, *TREM2* ablation was associated with increased Aβ pathology and formation of dystrophic neurites (another hallmark of AD, defined as neuronal processes characterized by accumulated cytoskeleton proteins and intraneuronal organelles [114, 115]. As such, in an independent study using 5xFAD mice, knockout of CD33 molecule/gene (*Cd33*), a transmembrane receptor protein, alleviated Aβ pathology and cognitive decline. However, these benefits were reversed by concurrent *Trem2* deletion [116]. Specifically, *Trem2* knockout exacerbated Aβ deposition and pathology, increased neurodegeneration and inflammation, and downregulated microglial phagocytosis-related genes in 5xFAD mice [116]. These findings highlight a functional interplay between CD33 and TREM2 in modulating microglial responses, Aβ clearance, and neuroprotection. Given the driving role of Aβ plaques in neurodegeneration, it can be argued that TREM2-mediated clearance of Aβ plaques indeed serves as a mechanism to avert AD-associated neurodegeneration. Moreover, these findings denote that TREM2 activation favors participation of microglia in the complement system (a part of innate immunity) and improves microglia lysosomal/phagosomal activity, all contributing to Aβ clearance. We hypothesize that other elusive mechanisms may exist, underpinning TREM2-mediated removal of Aβ plaques, however, their discoveries are pending on additional basic studies. For instance, in AD mice and in vitro models, mechanistically, it was shown that hippocampal upregulation of *TREM2* induced M2 (anti-inflammatory) polarization of microglia through activation of the phosphoinositide 3-kinase – AKT serine/threonine kinase 1 – factor forkhead box O3 protein (PI3K-AKT1-FoxO3a) signaling, thereby leading to inhibition of neuroinflammation (Fig. 1) [117]. However, its ablation was accompanied by inflammatory polarization of microglia and the release of proinflammatory cytokines, which worsened neuroinflammation and cognitive function [117].

Taken together, these findings revealed one of the underpinning mechanisms of TREM2-mediated suppression of neuroinflammation and improved cognitive function in AD mice. In detail, non-microglia studies have revealed that activated AKT1 directly phosphorylates FoxO3a, causing its inhibition. As a result, FoxO3a transcription factor is no longer able to transcriptionally upregulate proinflammatory cytokines such as *TNF*/TNF-α, interleukin-6 (*IL6*/IL-6), and *IL1B*/IL-1β, leading to inhibition of neuroinflammation [118–121].

In an independent study on AD mice, exercise induced *TREM2* upregulation in the hippocampus, which was shown to inhibit microglia-mediated neuroinflammation. However, TREM2/*TREM2* downregulation promoted microglia-mediated neuroinflammation [122, 123]. Though these findings are informative, they fail to elaborate on functional mechanisms underpinning TREM2-mediated suppression of neuroinflammation in the hippocampus. Moreover, exercise was introduced as a non-invasive approach to upregulate TREM2 expression and therefore therapeutically maneuver hippocampal neuroinflammation.

As aforementioned, *TREM2* gene mutations have been shown to be linked with AD [92]. In an early study, exome, genome, and Sanger sequencing techniques were utilized to explore *TREM2* gene variability in a cohort of 1092 patients with AD and 1107 individuals as the control group [124]. The *rs75932628* variant (causing R47H amino acid substitution) was also genotyped in other series of 1887 AD patients with 4061 controls [124]. Moreover, the expression of *TREM2* gene and its variants were explored in different brain regions in AD mice [124]. Exon 2 of the *TREM2* gene exhibited highest number of variants including 22 variant alleles and the *rs75932628* variant was the most common variant and significantly associated with AD prevalence [124]. In AD mice, *TREM2* expression levels differed from that of controls [124]. Altogether, these findings denote that genetic mutations in *TREM2* gene including the heterozygous *rs75932628* variant are closely associated with AD risk. Mechanistically, impaired containment of neuroinflammatory process is the main cause of increased AD risk under *TREM2* variants [125].

The current evidence largely supports that *TREM2* loss-of-function mutations favor Aβ pathology and AD development risk [125, 126], however, regarding tau pathology, controversial reports exist. In one study, it was reported that *TREM2* deficiency alleviated neuroinflammation and brain atrophy and protected against tau pathology and neurodegeneration in PS19 transgenic mice [127]. Therefore, in contrast to Aβ pathology, TREM2 and microglia activation might play pathological roles in tau pathology in AD, however, further studies are required to reveal which molecular mechanisms underscore such implications. In another study on Tau PS2APP mice, which recapitulates an AD state comprising of both Aβ plaque and tau pathology, *TREM2* deficiency was shown to favor accumulation, spread, and pathology of tau, ultimately, leading to increased brain atrophy [128]. To explain these findings, it is proposed that *TREM2* deficiency in the presence of Aβ plaques, promotes Aβ pathology, which contributes to accelerated phosphorylation and propagation of tau [128, 129]. Hence, the anti-inflammatory and neuroprotective effects of TREM2 appear to be largely contingent on the presence of Aβ pathology. In contrast, when tau pathology occurs in isolation, TREM2 activation may promote neuroinflammation and neurodegeneration, thereby contributing to AD progression. Supporting this, an independent study in 5xFAD mice demonstrated that TREM2 activation via the agonistic antibody AL002a exacerbated tauopathy and induced neurotoxicity without altering Aβ deposition [130]. Given that the underlying molecular mechanisms driving these divergent effects remain poorly understood, further mechanistic investigations are essential to clarify the context-dependent roles of TREM2 in AD pathogenesis.

In mouse models, *Trem2* ablation facilitated tau spreading and hippocampal pathology originating from the medial entorhinal cortex, which was accompanied by synaptic dysfunction and memory impairment [131]. In vitro, microglia-specific *Trem2* ablation similarly promoted interneuronal tau transfer and enhanced its release via microglial exosomes, thereby contributing to tau seeding and propagation [131]. Collectively, these findings indicate that TREM2 plays a protective role against tau pathology by limiting both its progression and exosomal dissemination, whereas Trem2 loss exacerbates these processes, accelerating AD progression. Consistently, additional studies have shown that *Trem2* knockout or expression of its *R47H* variant in mice enhanced tau spreading and seeding, and promoted tau aggregate formation, underscoring the pivotal role of microglial TREM2 in protecting against tau pathology and propagation [132, 133].

Longitudinal assessment of cerebrospinal fluid (CSF) soluble TREM2 (sTRM2) levels can provide insights into microglial activation and AD pathology, including Aβ and tau deposition [134]. Analyses using bioinformatic tools and the Alzheimer’s Disease Neuroimaging Initiative (ADNI) database revealed significant difference in CSF sTREM2 levels among AD subgroups and controls, and the rate of sTREM2 change was associated with cognitive performance in AD cohorts [134]. Notably, elevated CSF sTREM2 levels were linked to reduced tau tangles and Aβ plaque burden [134]. These findings suggest that CSF sTREM2 could serve as a potential biomarker for AD, with increased levels potentially exerting neuroprotective effects and slowing disease progression. Supporting this, the Chinese Alzheimer’s Biomarker and LifestylE (CABLE) study reported that higher CSF sTREM2 levels may mitigate *ApoE ε4*-associated Aβ pathology in cognitively healthy adults [135]. Nevertheless, the supporting mechanisms driving these associations remain to be fully elucidated and warrant further investigation.

In an independent study using transgenic mouse models overexpressing either human *TREM2* wild-type or the *R47H* variant, *TREM2* overexpression was found to reduce Aβ deposition and dystrophic neurite formation during early-stage AD, whereas overexpression of the R47H variant aggravated Aβ pathology during middle-stage AD [136]. RNA sequencing further revealed that *TREM2* overexpression attenuated the transcriptional signature and prevalence of disease-associated microglia (DAM) in early AD, while the *R47H* variant enhanced antigen-presenting pathways in mid-stage AD [136]. Collectively, these findings implicate TREM2 activation/expression in early AD pathogenesis, primarily through suppression of DAM activity. However, additional mechanistic studies are required to elucidate the molecular basis of TREM2-mediated modulation of DAM.

TREM2’s involvement in neuroinflammation and AD pathogenesis is thought to be partly mediated through the regulation of lipid metabolism [137, 138]. In a recent study using AD mouse models, TREM2 overexpression suppressed NF-κB-mediated neuroinflammation and slowed AD progression [138], potentially through modulation of lipid metabolism [138]. This anti-inflammatory effect was presumed to involve modulation of lipid metabolism. Although that study did not directly investigate TREM2’s functional role in lipid metabolic pathways, other evidence indicates that TREM2-deficient microglia exhibit impaired lipid metabolism, marked by the accumulation of intracellular lipid droplets [139]. These findings indicate that TREM2 may regulate key lipid-handling pathways in microglia, which in turn affect inflammatory responses. Therefore, in-depth mechanistic studies are required to elucidate the regulatory effects of TREM2 signaling on lipid metabolisms and related genes [140], particularly in the context of AD and neuroinflammatory processes.

## TREM2 in Parkinson’s associated neuroinflammation and neurodegeneration

As briefly discussed above, PD pathology involves intraneuronal accretion of α-Syn neurotoxic oligomers known as protofibrils in brain’s substantia nigra, thereby impairing neuronal homeostasis and inducing neuronal death and neurodegeneration of dopaminergic neurons [141–144].

Like AD, PD is bidirectionally linked with neuroinflammation [145–147]. In PD mice and in vitro models (recapitulating microglial exposure to α-Syn), *TREM2* ablation induced α-Syn-mediated inflammatory cascade in microglia in vitro, while its knockout was associated with α-Syn-mediated loss of dopaminergic neurons [148]. Collectively, these findings suggest that *TREM2* deficiency favors PD-associated neuroinflammation and neurodegeneration [148]. In contrast, *TREM2* overexpression was associated with suppression of neuroinflammation and prevention of neurodegeneration in dopaminergic neurons in PD mice (induced by methyl-phenyl-tetrahydropyridine [MPTP]) [88]. From the mechanistic viewpoint, TREM2 activation on microglia induces inhibition of the toll-like receptor 4 – TNF receptor-associated factor 6 – mitogen-activated protein kinase – nuclear factor kappa B (TLR4-TRAF6-MAPK-NF-κB) signaling axis, thereby inhibiting inflammatory response and neuroinflammation. In detail, the neuroinflammatory role of NF-κB involves its mediated transcriptional activation of proinflammatory genes such as cytokines and chemokines that are released from microglia, thus contributing to escalation of chronic inflammation, ultimately, neuroinflammation [149, 150]. Also, activation of the MAPK signaling is triggered by the TLR4-TRAF6 signaling, which induces phosphorylation of the MAPK kinase 3 (MKK3), 4, 6, and 7, leading to activation of the MAPK8/JNK1 (c-Jun N-terminal kinase) signaling and c-Jun activation (Fig. 1) [151–153]. c-Jun is a transcription factor that can combine with c-Fos factor to produce another transcription factor, known as activator protein 1 (AP-1) that translocates to the nucleus and upregulates proinflammatory gene transcriptions, thus inducing inflammation [154]. In sum, activation of the TLR4-TRAF6 axis could be linked with neuroinflammation via the NF-κB and MAPK pathways, and anti-neuroinflammatory role of TREM2 is largely attributed to modulation of these pathways. In an independent study, in PD mice and in vitro models, *TREM2* deficiency drived activation of the NF-κB signaling, leading to priming and activation of the NLR family, pyrin domain containing 3 (NLRP3) inflammasome, subsequently activation of proinflammatory cytokines, neuroinflammation, neurodegeneration of dopaminergic neurons, and pyroptosis (inflammatory cell death) [155]. The NLRP3 inflammasome can activate pro-caspase-1, thus producing caspase 1 that activates pro-IL-18 (interleukin 18) and pro-IL-1β (interleukin 1 beta) cytokines. After activation, these cytokines are released and induce inflammation, which could develop into neuroinflammation [156]. In support of this, multiple lines of evidence indicate that NLRP3 inflammasome can drive sterile neuroinflammation in the context of AD and PD as well as hemorrhagic stroke [157–160].

Altogether, these findings denote that *TREM2* deficiency is linked with microglia-mediated neurodegeneration and neuroinflammation in PD due to the TLR4-TRAF6-MAPK/NF-κB signaling activation. Nonetheless, further studies are required to disclose other pertinent mechanisms of TREM2-induced suppression of neuroinflammation in PD and related diseases.

As noted above, CSF’s sTREM2 levels may offer valuable insights on AD pathogenesis and emerging evidence suggests a similar relevance in PD pathogenesis [161]. In a recent study involving 219 PD patients and 100 controls, longitudinal and cross-sectional analyses using multivariable-adjusted models examined association between CSF sTREM2 levels and cognitive function [161]. Elevated CSF sTREM2 levels were linked to greater overall cognitive decline across PD clinical subgroups [161]. These findings support the potential of CSF sTREM2 measurement as a predictive tool for cognitive decline in PD, as well as an indicator of neuronal injury or damage [161]. Nevertheless, further research is required to clarify the pathological significance of increased CSF sTREM2 levels and their possible modulatory effects in PD.

## Conclusions and final remarks

Several key mechanisms have been proposed to demonstrate the protective and anti-neuroinflammatory role of TREM2 activation in microglia within the CNS. Overall, these mechanisms tend to be anti-neurodegenerative, promoting the retardation of neurodegeneration as observed in the context of both AD and PD. Despite some controversies, TREM2 is primarily recognized as an anti-neuroinflammatory and anti-neurodegenerative molecule. One such mechanism involves TREM2 enhancing the antigen-presenting capacity of microglia and promoting the generation of complement system components, as well as the lysosomal and phagosomal functions of microglia. These actions contribute to the increased clearance of Aβ plaques and the retardation of AD-associated neurodegeneration. As new research directions, the same trend of investigations should be applied for PD to expand our knowledge of the pathogenesis and associated neuroinflammation in greater details. Additionally, TREM2 activation triggers the PI3K-AKT1-FoxO3a signaling pathway, which inhibits neuroinflammation. In PD, TREM2 activation or overexpression in microglia also mitigates neuroinflammation and prevents neurodegeneration. These effects are largely attributed to the inhibition of the TLR4-TRAF6-MAPK/NF-κB signaling. Interestingly, but not unexpectedly, physical exercise has been shown to act as a non-invasive approach to activate TREM2, therefore promoting its protective effects in the context of both AD and PD. Importantly, TREM2’s role in AD tau pathology differs from its involvement in Aβ pathology, as some evidence suggests that TREM2 may exacerbate neurodegeneration and neuroinflammation in the context of tau-related pathology. Overall, our understanding of the molecular mechanisms and implications of microglial TREM2 in neurodegeneration and associated diseases is expanding. Further basic research is essential to elaborate on these findings, paving the way for future clinical studies and the development of effective translational approaches. Indeed, further studies are required to disclose other pertinent mechanisms and pathways underpinning TREM2-mediated inhibition of neuroinflammation in the context of PD, AD, and related diseases. Besides, contradictory roles of TREM2 in AD-associated neuroinflammation, particularly, in the presence of Aβ or tau pathologies or both require extensive exploration and basic/clinical studies. Also, elaborating on molecular mechanisms that underlie TREM2-mediated suppression of neuroinflammation in the hippocampus seems to be a requisite as this area has not been sufficiently covered by the current literature. Translational aspects of these findings also should be discussed thoroughly as they remain a matter of concern. Unfortunately, as we attempted throughout the manuscript to highlight the pros and cons of these findings as well as elucidating potential remaining points for further investigation by prospective studies, translational merits have largely remained untouched and unexplored. However, as we illustrated in the above sections, potential molecular targets have been exposed by current findings, which can be translated into practical intervention at both preclinical and clinical levels, pending adequate number of basic studies with prompt focus on molecular mechanisms as well as large-scale clinical studies. In other words, there is still a long path to go before establishing reliable molecular targeting techniques and drugs for intervention of these mechanisms for therapeutic purposes.
